# Promoting self-management behaviors in adolescents with type 1 diabetes, using digital storytelling: a pilot randomized controlled trial

**DOI:** 10.1186/s12902-022-00988-7

**Published:** 2022-03-22

**Authors:** Nahid Zarifsaniey, Masoomeh Otrodi Shirazi, Manoosh Mehrabi, Zahra Bagheri

**Affiliations:** 1 Department of E-Learning, Virtual School, Shiraz University of Medical Sciences, Neshat Avenue, 1846- 71345 Shiraz, Iran; 2 Department of Biostatistics, Faculty of Medicine, Shiraz University of Medical Sciences, Shiraz, Iran

**Keywords:** Type 1 diabetes, Adolescents, Training, Digital storytelling, Glycosylated hemoglobin, Self-management

## Abstract

**Background:**

This study aimed to assess the effects of digital storytelling on the self-management behavior of adolescents with type 1 diabetes (TID).

**Methods:**

In this pilot randomized controlled clinical trial, 60 adolescents with TID were randomly allocated into two parallel groups: intervention (training with digital storytelling method, *n* = 33) or control (training with a conventional method, *n* = 33). The primary outcome was assessing the Self-Management behavior of adolescents with TID (SMOD-A) at baseline and three months after the intervention.

**Results:**

The results revealed that digital storytelling could significantly improve self-management behaviors amongst adolescents with TID (*P* = 0.005). In contrast, in the control group, no significant changes were observed (*P* > 0.05). Furthermore, the mean score of Collaboration with Parents subscale was significantly higher in the digital storytelling group than in the control group after the intervention (*p* = 0.022). The results also showed that the level of Collaboration With Parents' subscale scores had a meaningful reverse relationship with the adolescent age after digital storytelling (*p* = 0.048). Repeated measures ANOVAs showed that there were significant main effects of time and group on collaboration with parents(*p* = 0.002) and goal subscales (*p* = 0.035). With respect to HbA1c levels, significant changes were not observed in any of the groups (*P* > 0.05).

**Conclusions:**

Digital storytelling is practicable and a potentially beneficial training modality for adolescents with TID.

**Trial registration:**

This trial was respectively registered.

ClinicalTrials.gov Identifier: IRCT20191220045828N1.

Date of registration: Oct 29. 2020.

**Supplementary Information:**

The online version contains supplementary material available at 10.1186/s12902-022-00988-7.

## Background

Type 1 diabetes (TID) is one of the most prevalent endocrine diseases among adolescents, and its prevalence is increasing globally [1]. Diabetes is a chronic condition that requires particular self-management behaviors throughout life. Self-management can improve patients' general health, active engagement in the care process, enhance the quality of life, and, ultimately, reduce chronic complications [2]. Studies revealed that self-management could be beneficial to adolescents in order to advance their knowledge and skills, leading to appropriate changes in behavior. Promoting self-management is one of the critical factors in controlling diabetes [3].

Self-management amongst adolescents is multidimensional and should include activities that youths and their parents can execute to care for the disease as well as collaboration with health care providers that assist them toward accepting full responsibility for managing their illness [4]. Self-management education can help youngsters with diabetes gain more independence, enabling them to learn that managing diabetes prevents critical and chronic complications and glycemic control [5].

Furthermore, research has shown that the rate of self-management in adolescents with diabetes is much lower than in adults; consequently, adolescents face more complications than adults [6].

Diabetes self-management education is an integral part of its management and should be taught to all diabetes patients. Several studies have examined different training modalities to promote self-management for tackling diabetes, including in-person training, text messaging [7], video [8], cellphone applications [2], etc. However, different results were achieved, and what is evident is that different educational methods are not equally effective; consequently, it is necessary to evaluate their effects.

On the other hand, new educational modalities have paved the way to develop new teaching methods using new technologies. Amongst them, digital storytelling seems to be an effective method to train adolescents. This modality can provide a robust structure to organize and present data to its audience, regardless of the limitations caused by the learners' age, education, or learning level [9]. In contrast to the traditional modalities, the storytelling approach incorporates memory-enhancing features, such as the story characters. The audience can search for these characters in his mind or even tries to associate with them. These factors can make the topic closer to the audience's mindset. The story's sequence can help them recall the storylines and engrave the underlying educational concepts in their long-term memory [10]. Consciousness and motivation stimulate attention, resulting in enhanced active learning and understanding of the subject. The audience's level of awareness in the traditional classes quickly declines after 10 min; however, in the storytelling method, the audience usually remains conscious and motivated [11], resulting in much better learning and understanding of the topic [12].

Storytelling is an approach that enables afflicted people with diseases, such as diabetes to understand their condition better and find the right self-management strategies. Storytelling can combine learning with improving coping skills, ultimately motivating patients to change their lifestyles [13].

Digital storytelling is a technology-based method in which the appropriate integration of a story with multimedia capabilities enables a more straightforward, broader, and more engaging transfer of educational concepts to facilitate active learning [10]. Today, this method is widely used for transferring concepts to adolescences. Several studies were conducted on the effectiveness of this method, including the ones on the effects of digital storytelling on the academic achievements of young people [12], anxiety in patients undergoing cardiac surgery [13], adolescents' social intelligence [10], anxiousness and coping strategies in children with cancer [14]; in addition to social skills amongst children by improving the sensory and cognitive awareness in autistic young people [11].

Although digital storytelling is a useful education tool for adolescents, this issue has received little attention in self-management training for adolescents with diabetes. Given that no research has been carried out in this context at Shiraz University of Medical Sciences; hence, the current research was performed to investigate the effects of digital storytelling on adolescents with TID and their self-management behaviors.

## Methods

### Design

This pilot randomized controlled trial was conducted to evaluate the feasibility of using digital storytelling for self-management education in diabetes adolescence. The study adheres to CONSORT 2010 guidelines (Additional file 1).

### Participants

#### Eligibility criteria for participants

Inclusion criteria were all adolescents with TID between the age of 12 and 18 who were using medication (insulin) and willing to participate in the study. The patients with any known physical or mental disabilities which interfered with their ability to complete questionnaires, use educational content, and answer the phone or send messages were excluded. Additionally, those who were using anti-anxiety or anti-depressant medication or had attended other educational programs or courses on diabetes in the preceding six months were eliminated. Further, all patients who expressed an unwillingness to continue participating in this study were omitted.

### Setting

The study population included adolescents with TID who attended diabetes clinics affiliated with Shiraz University of Medical Sciences from July 2018 till September 2019.

### Intervention

The intervention group received asynchronous digital diabetes self-management education, using storytelling and routine training. The study lasted three months, in which the adolescents in the intervention group received digital storytelling content after providing usual care and training. Then, every two weeks, participants received 10-min phone calls to follow up on the intervention. This program included assessing adolescents' comprehension of educational content, answering their questions, monitoring their progress, and encouraging them to practice self-management techniques. The adolescents were referred to the clinic three months after receiving the intervention and underwent a test post.

The digital content in this study is a training called "Diabetes and Bumblebee" designed and employed by the content development team, including an instructional designer, a pediatric endocrinologist, and one animation designer based on the nine-step content development model [15]. It features 35 min of animation depicting the daily life of a youngster with diabetes and providing necessary education. It is composed of a brief description of the disease, its symptoms and complications, treatment methods, the type of insulin, how to preserve insulin, the importance of nutrition and exercise in treating diabetes, and efficient communication and collaboration with parents and health care providers.

**Fig. 1:**
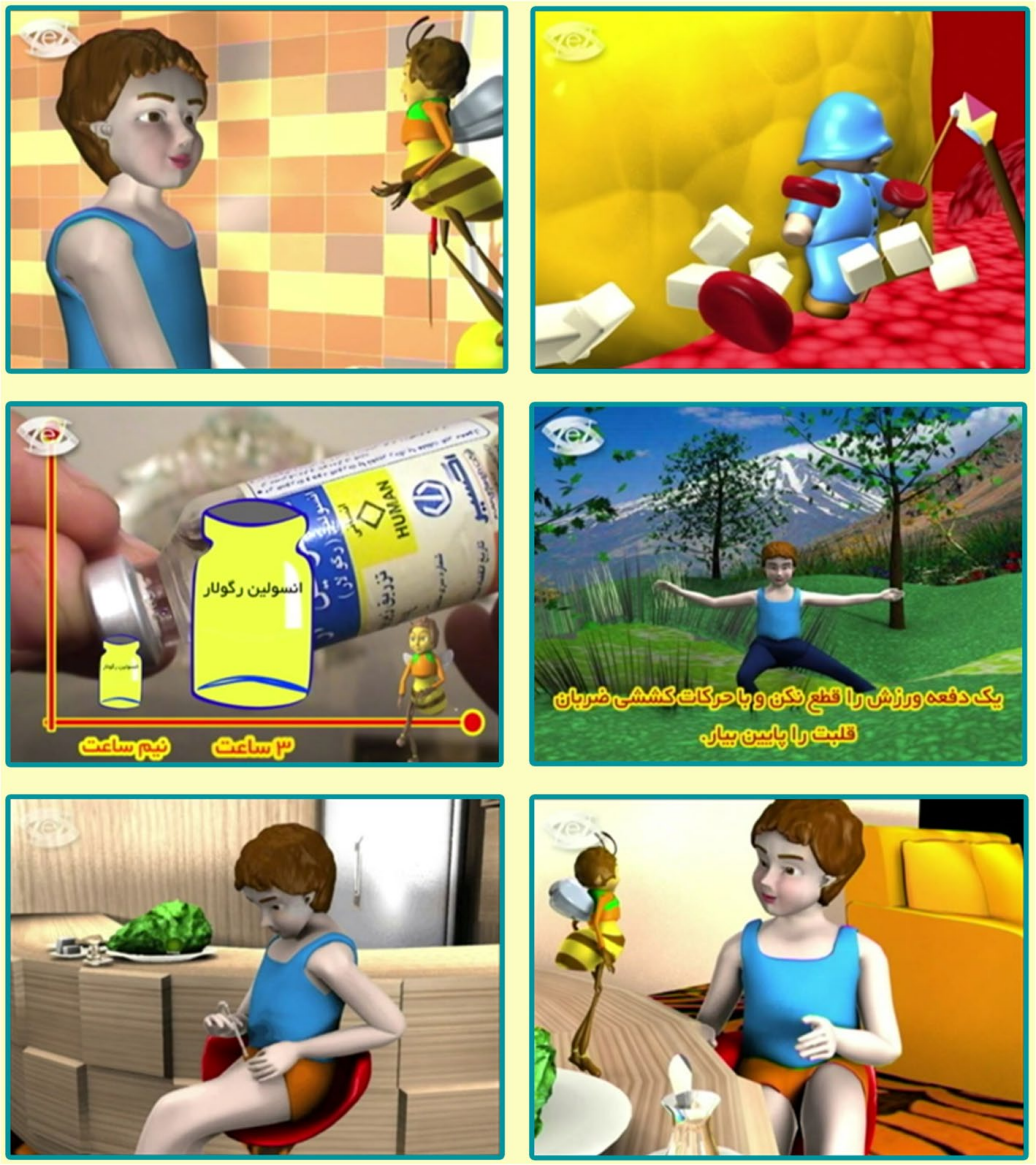
Screenshots of the digital storytelling DVD developed by the virtual school

The main cartoon character is called "Bumblebee," who explains the necessary points in a very simple and tangible language. The adolescent in our story shares his questions and has discussions about diabetes with his friend "Bumblebee." The Bumblebee also teaches him what youth with diabetes needs to know through storytelling. For example, an adolescent is shown how to inject insulin or where to do the injection (Fig. 1).

### Wait-list control group

The control group, on the other hand, received routine in-person group training. They were educated about the description of the disease, its symptoms, complications, treatment approaches, the type of insulin, how to preserve insulin, the importance of nutrition and exercise over a three-hour workshop, using PowerPoint and instructional video, and answering participants questions. The "Bumblebee" training DVD was also given to the control group at the end of the research.

### Outcomes

The primary outcome of the present study was to assess the Self-Management behavior at baseline and three months after interventions in both groups (intervention and control). The Self-Management behavior was evaluated according to the Self-Management of TID amongst adolescence, using (SMOD-A) questionnaire. The Persian version of the SMOD-A was used to check self-management behaviors in adolescents with TID. This version is a translation of the original self-management questionnaire by Schilling et al., which includes 48 items in 5 domains; Cooperation With Parents (the extent of parental involvement in diabetes management- 10 items), Diabetes Care Activities (the extent to which the adolescents perform key diabetes management activities -12 items), Diabetes Problem-Solving (the extent the adolescent following the diet and knows the amount of HbA1c -8 items), Diabetes Communication (how often does the adolescent communicate with parents, health care team, and friends about their diabetes -12 items), and Goal in Diabetes (more independent management of diabetes, prevention of uncontrolled blood glucose complications, participation in normal social activities with friends- 6 items). Each item is scored on a 4-point Likert scale ranging from 0 (never) to 3 (always). Schilling et al. reported excellent content validity for this questionnaire (0.89) and acceptable reliability of its subscales (a = 0.71–0.85) [16, 17]. Alaei Karahroody et al. also reported acceptable content validity (0.98) and test–retest reliability for the Persian version of this questionnaire (0.73) [16]. Higher scores are associated with better self-management behaviors. The Sum of the score was 0.144. Good self-management is shown by a higher than 70% total score. The participants had to spend 20 min to fill the questionnaire.

The secondary outcome was to assess the levels of HbA1c at baseline and three months after interventions in both groups (intervention and control). HbA1C was used to determine the average level of blood glucose. Glycosylated hemoglobin is a biological indicator for self-management in diabetes and is considered as the basis for treatment plans for patients with diabetes.

For adolescents without diabetes, the normal range for the HbA1c level is between 4 to 5.6%. In patients with diabetes, the level is 7% and higher regarding the ISPAD and ADA guidelines [18].

In this study, blood sampling and blood tests were conducted in the laboratory of a clinic at the research site. This process was supervised by one of the laboratory staff with sufficient knowledge and skills in this field.

### Sample size

The final sample size was determined based on the pilot study results we had conducted before the main study. The mean of total self-management score was considered for sample size determination which was 97.61 and 88.71 in the intervention and control group, respectively, with the pooled standard deviation of 12.48. Assuming type I error = 0.05, power = 0.8, and the following equation, the sample size was determined at 33 patients in each group. Including the 30 patients from the pilot study (15 in each group), we selected 18 other patients in each group to reach the required sample size. This calculation ${Z}_{1-\frac{\alpha }{2}}= 1.96$ is considered 1.96.

$$n=\frac{{({Z}_{1-\frac{\alpha }{2}} + {Z}_{1-\beta })}^{2}({S}_{1}^{2} + {S}_{2}^{2})}{{({\mu }_{1}-{\mu }_{2})}^{2}}$$

### Randomization

The eligible Participants were randomly assigned to the intervention and control groups based on permuted block randomization. Since the total sample size was 66 (33 in each group), the block size was six, and the number of blocks was eleven. We used random allocation software to generate the list of randomization. The samples were blinded to intervention and control groups. The randomization and blinding were conducted by an assistant researcher who was not involved in the research protocol.

### Blinding

In this single-blinded randomized trial, the samples were blinded to intervention and control groups.

### Statistical methods

The collected data was analyzed in SPSS-15 by using descriptive and inferential statistical tests. The demographic variables were analyzed using descriptive statistics (mean, SDs, frequencies, and percentages). Independent Samples T-Test and Chi-square test were used to compare the two groups' numerical and categorical demographic variables. Furthermore, to investigate whether the intervention can lead to significant changes in each group, Paired Sample T-TEST was used to compare the mean scores of self-management and HbA1c before and after the study.

To determine whether there was a significant difference between the two groups before the intervention, the mean scores of self-management and pre-HbA1c were compared by Independent Samples T-Test. For comparing the self-management scores and HbA1c after the study while controlling the effects of age and pre-HbA1c (which were significant between the two groups), and self-management scores before the intervention, analysis of covariance (ANCOVA) test was applied separately for each variable. To examine whether the change over time (from pre- to post measurements) in the intervention group differs from the control group, repeated measures analysis of variance test was used.

The Pearson correlation coefficient was used to assess the correlation between age and post-self-management scores, as well as HbA1c. *P*-value < 0.05 was considered to be statistically significant.

## Results

**Fig. 2:**
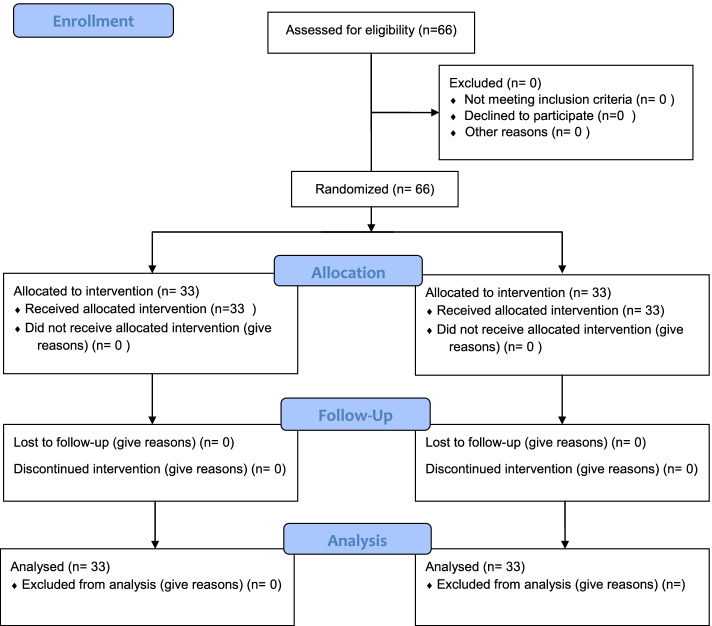
The patient’s recruitment flow diagram

The 66 recruited individuals were enrolled through convenience sampling, and eligible participants were randomized using random allocation software. The participants were blinded into two "parallel" groups; intervention (digital storytelling) and control (conventional intervention) groups (Fig. 2).

The study lasted between July 2018 and September 2019. All the randomized patients completed the trial and the follow-up assessment. Participants' demographic characteristics are shown in Table 1.

**Table 1:** Participants' demographic data in digital storytelling and control groups

Variables	Digital storytelling	Control	*P*-value
	Mean ± SD	Mean± SD	
**Age**	11.66±3.67	13.42±3.21	0.043^*^
**Gender**	N (%)	N (%)	
Girls	19(57.6)	22(66.7)	0.447^¶^
Boys	14(42.4)	11(33.3)	
**Elapsed time since diagnosis of type 1 diabetes**			
Less than 1 year	10(30.3)	6(18.2)	0.251^¶^
More than 1 year	23(69.7)	27(81.8)	
**Mother's Educational level**			
Under diploma	16(48.5)	19(57.6)	0.459^¶^
Diploma and above	17(51.5)	14(42.4)	
**Father's Educational level**			
Under diploma	18(54.5)	15(39.4)	0.218^¶^
Diploma and above	13(45.5)	20(60.6)	

^*^: *P*-value based on independent sample t-test

^¶^: *P*-value based on Chi-square test

As shown above, the mean age of individuals in the control group was significantly higher than those in the digital storytelling group. However, gender, time of diagnosis, parents' education level did not differ significantly between the groups.

Table 2 shows the mean score of the self-management of TID amongst adolescence (SMOD-A) subscales and HbA1c in the intervention and control groups before and after the intervention.

**Table 2:** Within- and between-group comparison of the self-management subscale scores before and after the intervention

Variables	Digital storytelling			Control				
	Before	After		Before	After		Between-group comparison	
	Mean ± SD	Mean ± SD	*p*-value^w^	Mean ± SD	Mean ±SD	*p*-value^w^	*p*-value^b^	*p*-value^a^
Collaboration With Parents	23.78±5.17	26.06±5.43	<0.001	22.69±5.81	22.36±6.05	0.620	0.423	0.022
Diabetes Care Activities	17.15±6.75	16.78±4.42	0.739	17.78±5.67	18.00±4.98	0.789	0.680	0.058
Diabetes Problem-Solving	14.15±4.71	15.36±5.07	0.155	14.93±3.52	15.12±3.07	0.746	0.445	0.593
Diabetes Communication	24.57±9.22	26.69±9.06	0.015	23.93±6.61	24.54±7.59	0.464	0.749	0.663
Goals	13.21±3.37	15.21±3.07	<0.001	14.78±3.06	15.33±2.21	0.241	0.051	0.356
Total	92.87±21.28	100.12±18.2	0.005	94.15±18.02	95.36±17.05	0.574	0.794	0.527
HbA1c	8.76±2.14	8.75±1.97	0.991	9.97±2.03	9.20±2.31	0.166	0.030	0.419

*p*-value^w^: within-group comparison in each interventional and control group

*p*-value^b^: between-group comparison before the intervention

*p*-value^a^: between-group comparison after the intervention based on ANCOVA test with controlling the effect of age, HbA1c and pre self-management scores

Our findings revealed that there was no significant difference between digital storytelling and control groups in terms of self-management scores in all subscales and total sores before the intervention, except for HbA1c (the column *P*-value^b^). Moreover, in the digital storytelling group, the mean Collaboration With Parents scores increased significantly from 23.78 ± 5.17 to 26.06 ± 5.43 after the intervention (*p*-value < 0.001). Additionally, the mean score of the Diabetes Communication dimension increased significantly (from 24.57 ± 9.22 before the intervention to 26.69 ± 9.06 after the intervention, *p*-value = 0.015). There was a two-unit increase in the mean score of the Goal dimension (*p*-value < 0.001). In addition, the total score of self-management raised significantly from 92.87 ± 21.28 before the intervention to 100.12 ± 18.2 after the intervention (*p*-value = 0.005).

The ANCOVA test was applied since we intended to control the effect of age and pre-HbA1c, which significantly differed between the intervention and control groups and the self-management scores in each dimension before the study. Our results showed that there was no significant difference between the groups after the study in all dimensions of self-management, total score, and HbA1c, except for the Collaboration With Parents subscales; the mean score of Collaboration With Parents was significantly higher in the digital storytelling group as opposed to the control group (26.06 ± 5.43 *vs.* 22.36 ± 6.05, *p*-value = 0.022). This implied that our intervention had a beneficial effect on this subscale of self-management. In addition, the repeated measures ANOVA was used to evaluate the interaction between time and groups (Table 3).

**Table 3:** The results of repeated measures ANOVA for each subscale of self-management subscales

	Digital storytelling		Control		*p*-value		
	Before	After	Before	After			
	Mean ± SD	Mean ± SD	Mean ± SD	Mean ± SD	Time	Group	Time by group interaction
Collaboration With Parents	23.78±5.17	26.06±5.43	22.69±5.81	22.36±6.05	0.021	0.075	0.002
Diabetes Care Activities	17.15±6.75	16.78±4.42	17.78±5.67	18.00±4.98	0.910	0.439	0.668
Diabetes Problem-Solving	14.15±4.71	15.36±5.07	14.93±3.52	15.12±3.07	0.169	0.763	0.307
Diabetes Communication	24.57±9.22	26.69±9.06	23.93±6.61	24.54±7.59	0.022	0.473	0.196
Goals	13.21±3.37	15.21±3.07	14.78±3.06	15.33±2.21	<0.001	0.195	0.035
Total	92.87±21.28	100.12±18.2	94.15±18.02	95.36±17.05	0.010	0.064	0.688
HbA1c	8.76±2.14	8.75±1.97	9.97±2.03	9.20±2.31	0.283	0.030	0.289

Time by group interaction was significant for collaboration with parents and goal subscales; hence, we could not rely on time and group effects. Also, there were significant main effects of time on the diabetes communication subscale and total score. These results imply that the subscale scores increased significantly from pre- to post-measurements. Furthermore, only the effect of the group was significant for HbA1c, with the magnitude of HbA1c being considerably higher in the digital storytelling group compared to the control group. To compare the effects of the intervention on the intervention group's self-management scores as opposed to the control group while controlling the baseline measurements, we focused on ANCOVA results. Table 4 presents the Pearson correlation between age and self-management scores along with HbA1c after the intervention in the digital storytelling group.

**Table 4:** Correlation coefficient between age and post-self-management scores along with post HbA1c in the digital storytelling group

	Collaboration With Parents	Diabetes care Activities	Diabetes Problem-Solving	Diabetes COmmunication	Goals	Total	HbA1c
	r (*p*-value)	r (*p*-value)	r (*p*-value)	r (*p*-value)	r (*p*-value)	r (*p*-value)	r (*p*-value)
Age	-0.347(0.048)	-0.208(0.246)	0.082(0.650)	-0.005(0.978)	0.125(0.488)	-0.112(0.533)	0.156(0.386)

It can be observed that there was only a significant relationship between age and post Collaboration with parents' scores; the increment of age led to the reduction of post Collaboration with parents scores (*r* = -0.347, *p*-value = 0.048). It should be noted that the relationship of the other baseline characteristics, such as mother and father education levels and adolescents' gender were also investigated, but no significant relation was observed. The results are not shown here due to space limitations.

It should be noted that no harm or unintended effects were observed in either group.

## Discussion

The present study revealed that self-management education through digital storytelling improved overall self-management behavior as well as Collaboration With Parents, Diabetes Communication, and Goals subscales amongst adolescents with TID compared to before the training. Moreover, the results showed that the score for self-management in the digital storytelling group significantly increased only in Collaboration With Parents subscale compared to the control group. Collaboration With Parents is a dynamic process in which adolescents and their parents share responsibility and actions for managing their disease and general well-being.

Parental participation in diabetes care must be balanced with adolescent self-management abilities. Excessive or inadequate involvement, according to research, may have a harmful influence on teenage self-management behaviors and competency [18]. The upsurge in the present study revealed that the adolescents were able to create a proper balance in the dividing caring responsibilities between parents and themselves, which ultimately led to more independence [19].

There are several reasons why digital storytelling can lead to superior performance. Compared to the conventional approach, storytelling provides a more exciting and enjoyable experience for youths, given their general interest in stories [9]. Thus, these advantages might result in greater effectiveness of training through the storytelling method.

On the other hand, patients in the control group underwent only one 2-h group educational session and a pamphlet at the end of the class. After this training, the teenagers did not receive any training until their next visit (three months later). Many studies have been conducted on the effectiveness of lecture training amongst youth patients. Most of which emphasize that lecture-based training can have little effect on long-term learning and the quality of self-management in young patients compared to active and attractive learning methods, such as digital storytelling. This method serves as one of the most fundamental methods to subliminally transfer concepts to audiences, especially adolescents [10]. Other research in this area also emphasizes the fact that training through digital storytelling method can have a significant effect compared to conventional methods by improving patients' healthy behaviors [19], lowering blood pressure [13], managing diabetes mellitus [20], and psychological well-being [12].

In addition, we used the asynchronous method to promote learning in our research. This method allows patients to interact with the story and its educational message and enable them to learn at their desired time, place, and pace. This finding is also in line with the results revealing the effects of asynchronous learning on knowledge, activation, self-management, and self-efficacy in patients with diabetes [21]. Therefore, asynchronous digital storytelling allows patients to learn at their own desired time, place, and learning pace to reach mastery in learning and self-management behaviors.

On the other hand, there was no synchronous communication between the health care providers and the adolescents in our study. Research has shown that supportive and consistent communication between health care professionals and adolescents with TID plays an essential role in improving self-management abilities [22]. As a result of the asynchronous nature of this type of intervention, the digital storytelling technique did not fully enhance patients' abilities in the Diabetes Care Activities, Diabetes Problem-Solving, and Goals subscales. It seems integrating digital storytelling with synchronous training with the health care providers leads to improved self-management in diabetes adolescence.

We also examined other factors that could potentially affect the results of our study. This study showed a significant reverse relationship between the age of the adolescents and collaboration with parents' scores after digital storytelling, while this relationship was not significant in the control group. The research also showed that as teenagers grow older, they become more independent for self-management through digital storytelling methods. Thus, cooperation and dividing responsibilities in self-management go gradually towards independence as adolescents get older [5].

Furthermore, the results showed that parents' education level had no significant relationship with the rate of change in self-management behaviors in adolescents with TID in any study group. Nowadays, the ever-growing activities of mass media and social networks have raised parents' awareness of various diseases (regardless of their level of education); hence, their compassion and rapid response have also increased [23]. This development can be considered as the reason for the lack of any significant relationship between parental education and the rates of change in self-management behaviors in adolescents with TID.

The results also showed that there was no significant relationship between the adolescents' gender and the rates of change in their self-management behaviors in both groups. The results of a study by Vasli & Eshghbaz [24] similarly showed no significant relationship between gender and self-management behavior in 7–14-year-old teenagers with TID. However, Martinez et al. [25] reported that self-management behavior in female adolescents with diabetes was better than in males. Of course, one should not ignore the role of context and environment in adolescents' self-care behaviors.‬‬‬‬‬‬‬‬‬‬‬‬‬‬‬‬‬‬‬‬‬‬‬‬‬‬‬‬‬‬‬‬‬‬‬‬‬‬‬‬‬‬‬‬‬‬‬‬‬‬‬‬‬‬‬‬‬‬‬‬‬‬‬‬‬‬‬‬‬‬‬‬‬‬‬‬‬‬‬‬‬‬‬‬‬‬‬‬‬‬‬‬‬‬‬‬‬‬‬‬‬‬‬‬‬‬‬‬‬‬‬‬‬‬‬‬‬‬‬‬‬‬‬‬‬‬‬‬‬‬‬‬‬‬‬‬‬‬‬‬‬‬‬‬‬‬‬‬‬‬‬‬‬‬‬‬‬‬‬‬‬‬‬‬‬‬‬‬‬‬‬‬‬‬‬‬‬‬‬‬‬‬‬‬‬‬‬‬‬‬‬‬‬‬‬‬‬‬‬‬‬‬‬‬‬‬‬‬‬‬‬‬‬‬‬‬‬‬‬‬‬‬‬‬‬‬‬‬‬‬‬‬‬‬‬‬‬‬‬‬‬‬‬‬‬‬‬‬‬‬‬‬‬‬‬‬‬‬‬‬‬‬‬‬‬‬‬‬‬‬‬‬‬‬‬‬‬‬‬‬‬‬‬‬‬‬‬‬‬‬‬‬‬‬‬‬‬‬‬‬‬‬‬‬‬‬‬‬‬‬‬‬‬‬‬‬‬‬‬‬‬‬‬‬‬‬‬‬‬‬‬‬‬‬‬‬‬‬‬‬‬‬‬‬‬‬‬‬‬‬‬‬‬‬‬‬‬‬‬‬‬‬‬‬‬‬‬‬‬‬‬‬‬‬‬‬‬‬‬‬‬‬‬‬‬‬‬‬‬‬‬‬‬‬‬‬‬‬‬‬‬‬‬‬‬‬‬‬‬‬‬‬‬‬‬‬‬‬‬‬‬‬‬‬‬‬‬‬‬‬‬‬‬‬‬‬‬‬‬‬‬‬‬‬‬‬‬‬‬‬‬‬‬‬‬‬‬‬‬‬‬‬‬‬‬‬‬‬‬‬‬‬‬‬‬‬‬‬‬‬‬‬‬‬‬‬‬‬‬‬‬‬‬‬‬‬‬‬‬‬‬‬‬‬‬‬‬‬‬‬‬‬‬‬‬‬‬‬‬‬‬‬‬‬‬‬‬‬‬‬‬‬‬‬‬‬‬‬‬‬‬‬‬‬‬‬‬‬‬‬‬‬‬‬‬‬‬‬‬‬‬‬‬‬‬‬‬‬‬‬‬‬‬‬‬‬‬‬‬‬‬‬‬‬‬‬‬‬‬‬‬‬‬‬‬‬‬‬‬‬‬‬‬‬‬‬‬‬‬‬‬‬‬‬‬‬‬‬‬‬‬‬‬‬‬‬‬‬‬‬‬‬‬‬‬‬‬‬‬‬‬‬‬‬‬‬‬‬‬‬‬‬‬‬‬‬‬‬‬‬‬‬‬‬‬‬‬‬‬‬‬‬‬‬‬‬‬‬‬‬‬‬‬‬‬‬‬‬‬‬‬‬‬‬‬‬‬‬‬‬‬‬‬‬‬‬‬‬‬‬‬‬‬‬‬‬‬‬‬‬‬‬‬‬‬‬‬‬‬‬‬‬‬‬‬‬‬‬‬‬‬‬‬‬‬‬‬‬‬‬‬‬‬‬‬‬‬‬‬‬‬‬‬‬‬‬‬‬‬‬‬‬‬‬‬‬‬‬‬‬‬‬‬‬‬‬‬‬‬‬‬‬‬‬‬‬‬‬‬‬‬‬‬‬‬‬‬‬‬‬‬‬‬‬‬‬‬‬‬‬‬‬‬‬‬‬‬‬‬‬‬‬‬‬‬‬‬‬‬‬‬‬‬‬‬‬‬‬‬‬‬‬‬‬‬‬‬‬‬‬‬‬‬‬‬‬‬‬‬‬‬‬‬‬‬‬‬‬‬‬‬‬‬‬‬‬‬‬‬‬‬‬‬‬‬‬‬‬‬‬‬‬‬‬‬‬‬‬‬‬‬‬‬‬‬‬‬‬‬‬‬‬‬‬‬‬‬‬‬‬‬‬‬‬‬‬‬‬‬‬‬‬‬‬‬‬‬‬‬‬‬‬‬‬‬‬‬‬‬‬‬‬‬‬‬‬‬‬‬‬‬‬‬‬‬‬‬‬‬‬‬‬‬‬‬‬‬‬‬‬‬‬‬‬‬‬‬‬‬‬‬‬‬‬‬‬‬‬‬‬‬‬‬‬‬‬‬‬‬‬‬‬‬‬‬‬‬‬‬‬‬‬‬‬‬‬‬‬‬‬‬‬‬‬‬‬‬‬‬‬‬‬‬‬‬‬‬‬‬‬‬‬‬

Moreover, the results of this study suggested that no significant relationship was observed concerning the rate of change in the level of HbA1c in neither of the groups. However, Azizi et al. [26] showed that self-management training with a 3-month follow-up through mobile services significantly reduced HbA1c in adolescents with TID. This is in contrast to the results of the present study and highlights the importance of long-term follow-up.‬‬‬‬‬‬‬‬‬‬‬‬‬‬‬‬‬‬‬‬‬‬‬‬‬‬‬‬‬‬‬‬‬‬‬‬‬‬‬‬‬‬‬‬‬‬‬‬‬‬‬‬‬‬‬‬‬‬‬‬‬‬‬‬‬‬‬‬‬‬‬‬‬‬‬‬‬‬‬‬‬‬‬‬‬‬‬‬‬‬‬‬‬‬‬‬‬‬‬‬‬‬‬‬‬‬‬‬‬‬‬‬‬‬‬‬‬‬‬‬‬‬‬‬‬‬‬‬‬‬‬‬‬‬‬‬‬‬‬‬‬‬‬‬‬‬‬‬‬‬‬‬‬‬‬‬‬‬‬‬‬‬‬‬‬‬‬‬‬‬‬‬‬‬‬‬‬‬‬‬‬‬‬‬‬‬‬‬‬‬‬‬‬‬‬‬‬‬‬‬‬‬‬‬‬‬‬‬‬‬‬‬‬‬‬‬‬‬‬‬‬‬‬‬‬‬‬‬‬‬‬‬‬‬‬‬‬‬‬‬‬‬‬‬‬‬‬‬‬‬‬‬‬‬‬‬‬‬‬‬‬‬‬‬‬‬‬‬‬‬‬‬‬‬‬‬‬‬‬‬‬‬‬‬‬‬‬‬‬‬‬‬‬‬‬‬‬‬‬‬‬‬‬‬‬‬‬‬‬‬‬‬‬‬‬‬‬‬‬‬‬‬‬‬‬‬‬‬‬‬‬‬‬‬‬‬‬‬‬‬‬‬‬‬‬‬‬‬‬‬‬‬‬‬‬‬‬‬‬‬‬‬‬‬‬‬‬‬‬‬‬‬‬‬‬‬‬‬‬‬‬‬‬‬‬‬‬‬‬‬‬‬‬‬‬‬‬‬‬‬‬‬‬‬‬‬‬‬‬‬‬‬‬‬‬‬‬‬‬‬‬‬‬‬‬‬‬‬‬‬‬‬‬‬‬‬‬‬‬‬‬‬‬‬‬‬‬‬‬‬‬‬‬‬‬‬‬‬‬‬‬‬‬‬‬‬‬‬‬‬‬‬‬‬‬‬‬‬‬‬‬‬‬‬‬‬‬‬‬‬‬‬‬‬‬‬‬‬‬‬‬‬‬‬‬‬‬‬‬‬‬‬‬‬‬‬‬‬‬‬‬‬‬‬‬‬‬‬‬‬‬‬‬‬‬‬‬‬‬‬‬‬‬‬‬‬‬‬‬‬‬‬‬‬‬‬‬‬‬‬‬‬‬‬‬‬‬‬‬‬‬‬‬‬‬‬‬‬‬‬‬‬‬‬‬‬‬‬‬‬‬‬‬‬‬‬‬‬‬‬‬‬‬‬‬‬‬‬‬‬‬‬‬‬‬‬‬‬‬‬‬‬‬‬‬‬‬‬‬‬‬‬‬‬‬‬‬‬‬‬‬‬‬‬‬‬‬‬‬‬‬‬‬‬‬‬‬‬‬‬‬‬‬‬‬‬‬‬‬‬‬‬‬‬‬‬‬‬‬‬‬‬‬‬‬‬‬‬‬‬‬‬‬‬‬‬‬‬‬‬‬‬‬‬‬‬‬‬‬‬‬‬‬‬‬‬‬‬‬‬‬‬‬‬‬‬‬‬‬‬‬‬‬‬‬‬‬‬‬‬‬‬‬‬‬‬‬‬‬‬‬‬‬‬‬‬‬‬‬‬‬‬‬‬‬‬‬‬‬‬‬‬‬‬‬‬‬‬‬‬‬‬‬‬‬‬‬‬‬‬‬‬‬‬‬‬‬‬‬‬‬‬‬‬‬‬‬‬‬‬‬‬‬‬‬‬‬‬‬‬‬‬‬‬‬‬‬‬‬‬‬‬‬‬‬‬‬‬‬‬‬‬‬‬‬‬‬‬‬‬‬‬‬‬‬‬‬‬‬‬‬‬‬‬‬‬‬‬‬‬‬‬‬‬‬‬‬‬‬‬‬‬‬‬‬‬‬‬‬‬‬‬‬‬‬‬‬‬‬‬‬‬‬‬‬‬‬‬‬‬‬‬‬‬‬‬‬‬‬‬‬‬‬‬‬‬‬‬‬‬‬‬‬‬‬‬‬‬‬‬‬‬‬‬‬‬‬‬‬‬‬‬‬‬‬‬‬‬‬‬‬‬‬‬

This study had several limitations. First, it was performed in only one center; hence, it has to be executed after the intervention on a wider scale for its results to be applicable as general. Furthermore, only short-term effects were investigated. So, the results cannot be generalized to all adolescents with TID. Therefore, long-term follow-up is warranted.

Future research recommends that diabetes self-management training through digital storytelling be administered for a much longer time and with a larger sample size to achieve a more accurate measurement of the level of HA1c and self-management behaviors in patients before and after the training course.

## Conclusion

Current study results prove that diabetes self-management training through digital storytelling can significantly improve adolescents with TID on self-management behaviors. However, there was no difference between the level of HbA1c in adolescence before and after the training.

## Supplementary Information

**Additional file 1.**

## Data Availability

The data that support the findings of this study are available from the corresponding author on request.
