# Supplementary material for: Promoting self-management behaviors in adolescents with type 1 diabetes, using digital storytelling: a pilot randomized controlled trial
Source: BMC Endocr Disord. 2022 Mar 22;22:74. doi: 10.1186/s12902-022-00988-7 (PMC8941790; doi:10.1186/s12902-022-00988-7)
Supplement: Supplementary file 1 — Additional file 1. [file 12902_2022_988_MOESM1_ESM.doc]

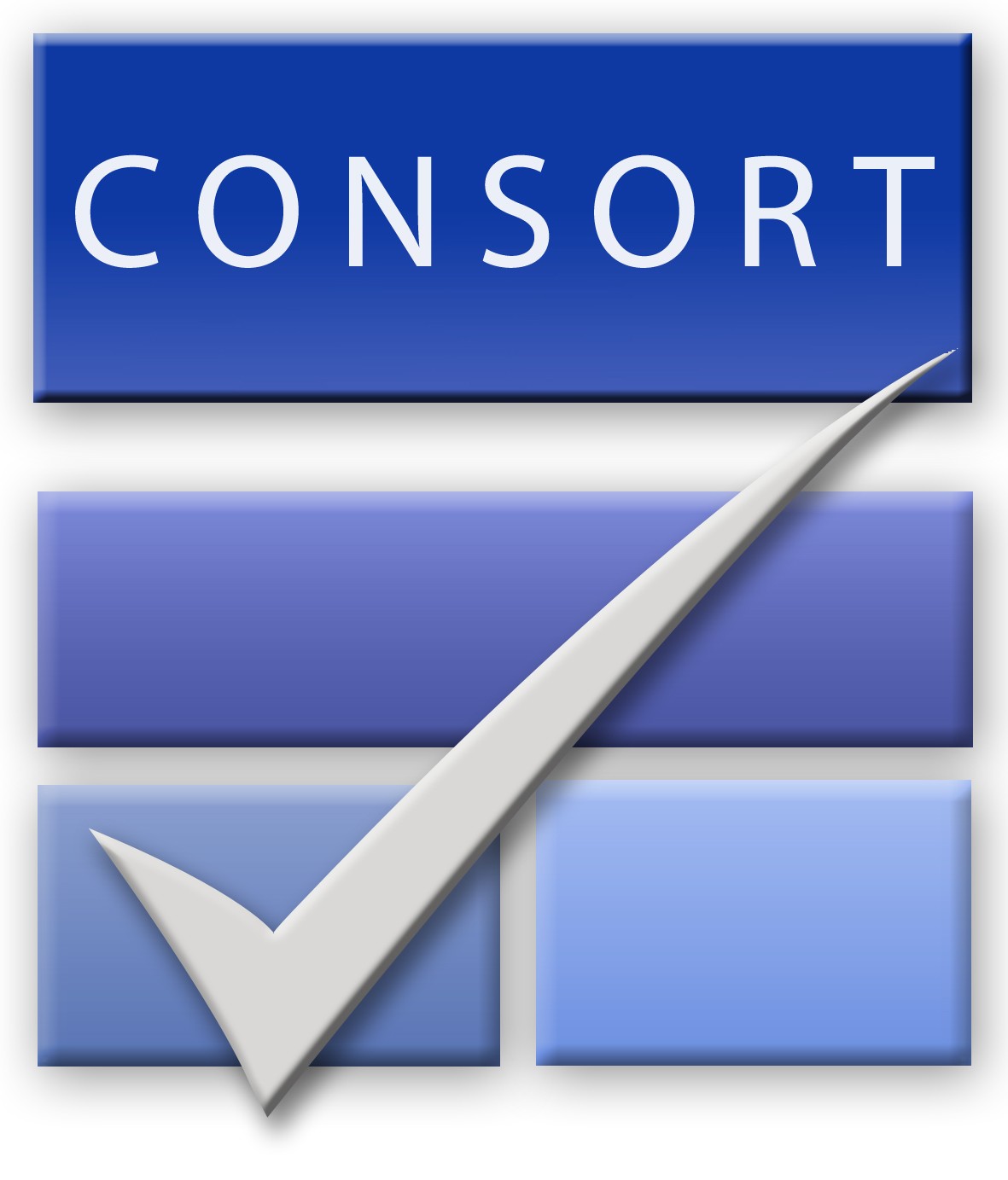
CONSORT 2010 checklist of information to include when reporting a randomised trial*

| Section/Topic | Item No | Checklist item | Reported on page No |
| --- | --- | --- | --- |
| Title and abstract | | | |
|  | 1a | Identification as a randomised trial in the title | Title  Page 1  paragraph1 |
|  | 1b | Structured summary of trial design, methods, results, and conclusions (for specific guidance see CONSORT for abstracts) | Abstract  Page2, paragraphs 2,3,4, 5 |
| Introduction | | | |
| Background and objectives | 2a | Scientific background and explanation of rationale | Introduction Page 4  Paragraphs 1,2  Page 5  Paragraphs  1 |
|  | 2b | Specific objectives or hypotheses | Introduction Page 5,  Paragraph 2 |
| Methods | | | |
| Trial design | 3a | Description of trial design (such as parallel, factorial) including allocation ratio | Methods Page 5 paragraph 3 |
|  | 3b | Important changes to methods after trial commencement (such as eligibility criteria), with reasons | N/A |
| Participants | 4a | Eligibility criteria for participants | Methods Page 6  Paragraph 1 |
|  | 4b | Settings and locations where the data were collected | Page 6  Paragraph 2 |
| Interventions | 5 | The interventions for each group with sufficient details to allow replication, including how and when they were actually administered | Methods Page 6  Paragraph 3  Page 7  Paragrah1  Page 9 Paragraph 1, 2 |
| Outcomes | 6a | Completely defined pre-specified primary and secondary outcome measures, including how and when they were assessed | Methods Page 9  Paragraph 3  Page 10  Paragraph 1,2 |
|  | 6b | Any changes to trial outcomes after the trial commenced, with reasons | N/A |
| Sample size | 7a | How sample size was determined | Methods Page 10  Paragraph 3 |
|  | 7b | When applicable, explanation of any interim analyses and stopping guidelines | N/A |
| Randomisation: |  |  | Methods Page 11  Paragraph 1 |
| Sequence generation | 8a | Method used to generate the random allocation sequence |  |
|  | 8b | Type of randomisation; details of any restriction (such as blocking and block size) | Methods Page 11  Paragraph 1 |
| Allocation concealment mechanism | 9 | Mechanism used to implement the random allocation sequence (such as sequentially numbered containers), describing any steps taken to conceal the sequence until interventions were assigned | Methods Page 11  Paragraph 1 |
| Implementation | 10 | Who generated the random allocation sequence, who enrolled participants, and who assigned participants to interventions | Page 11  Paragraph 1 |
| Blinding | 11a | If done, who was blinded after assignment to interventions (for example, participants, care providers, those assessing outcomes) and how | Methods Page 11  Paragraph 1 |
|  | 11b | If relevant, description of the similarity of interventions | N/A |
| Statistical methods | 12a | Statistical methods used to compare groups for primary and secondary outcomes | Methods Page 11  Paragraph 2 |
|  | 12b | Methods for additional analyses, such as subgroup analyses and adjusted analyses | Page 11  Paragraph 2 |
| Result | | | |
| Participant flow (a diagram is strongly recommended) | 13a | For each group, the numbers of participants who were randomly assigned, received intended treatment, and were analysed for the primary outcome | Result  Page 12  Paragraph 1  Page 14  Paragraph 1 |
|  | 13b | For each group, losses and exclusions after randomisation, together with reasons | Result  Page 14 Paragraph 1 |
| Recruitment | 14a | Dates defining the periods of recruitment and follow-up | Result  Page 14 Paragraph 1 |
|  | 14b | Why the trial ended or was stopped | N/A |
| Baseline data | 15 | A table showing baseline demographic and clinical characteristics for each group | Result  Page 14  Table 1 |
| Numbers analysed | 16 | For each group, number of participants (denominator) included in each analysis and whether the analysis was by original assigned groups | Result  Page 14  Table 1 |
| Outcomes and estimation | 17a | For each primary and secondary outcome, results for each group, and the estimated effect size and its precision (such as 95% confidence interval) | Result  Page 15  Table 2  Page 16  Paragraph 1 |
|  | 17b | For binary outcomes, presentation of both absolute and relative effect sizes is recommended | N/A |
| Ancillary analyses | 18 | Results of any other analyses performed, including subgroup analyses and adjusted analyses, distinguishing pre-specified from exploratory | Result  Page 16  Table 3  Page 17  Paragraph 1 |
| Harms | 19 | All important harms or unintended effects in each group (for specific guidance see CONSORT for harms) | Result  Page 17  Paragraph 2 |
| Discussion | | | |
| Limitations | 20 | Trial limitations, addressing sources of potential bias, imprecision, and, if relevant, multiplicity of analyses | Discussion Page 20  Paragraph 2 |
| Generalisability | 21 | Generalisability (external validity, applicability) of the trial findings | N/A |
| Interpretation | 22 | Interpretation consistent with results, balancing benefits and harms, and considering other relevant evidence | Discussion Page 17- 20 |
| Other information | | | Abstract  Page 2  Paragraph 5 |
| Registration | 23 | Registration number and name of trial registry |  |
| Protocol | 24 | Where the full trial protocol can be accessed, if available | N/A |
| Funding | 25 | Sources of funding and other support (such as supply of drugs), role of funders | Page 21  Paragraph 5 |

*We strongly recommend reading this statement in conjunction with the CONSORT 2010 Explanation and Elaboration for important clarifications on all the items. If relevant, we also recommend reading CONSORT extensions for cluster randomised trials, non-inferiority and equivalence trials, non-pharmacological treatments, herbal interventions, and pragmatic trials. Additional extensions are forthcoming: for those and for up to date references relevant to this checklist, see [www.consort-statement.org](http://www.consort-statement.org/).
